# *catena*-Poly[[diaqua­(2,2′-bipyridine-κ^2^ *N*,*N*′)nickel(II)]-μ-biphenyl-2,2′-dicarboxyl­ato-κ^2^ *O*:*O*′]

**DOI:** 10.1107/S1600536808036866

**Published:** 2008-11-13

**Authors:** Zhe An, Xian-Chun Niu

**Affiliations:** a School of Chemistry and Life Science, Maoming University, Maoming 525000, People’s Republic of China; b School of Chemical and Environmental Engineering, Maoming University, Maoming 525000, People’s Republic of China

## Abstract

In the title compound, [Ni(C_14_H_8_O_4_)(C_10_H_8_N_2_)(H_2_O)_2_]_*n*_, the Ni^II^ atom is coordinated in a slightly distorted octa­hedral geometry by two water mol­ecules, two N atoms from a 2,2′-bipyridine ligand and two O atoms from the carboxyl­ate groups of two 2,2′-biphenyl­dicarboxyl­ate (2,2′-dpa) ligands. The 2,2′-dpa ligand acts as a bridge between neighbouring Ni^II^ atoms, forming one-dimensional coordination polymers along [100]. The coordinated water mol­ecules form hydrogen bonds to the carboxyl­ate O atoms of 2,2′-dpa within the same coordination polymer, and one O—H⋯π inter­action is also formed to 2,2′-dpa.

## Related literature

For other metal–organic frameworks containing 2,2′-dpa, see: Rueff *et al.* (2003 ▶); Wang *et al.* (2006 ▶); Xu *et al.* (2006 ▶).
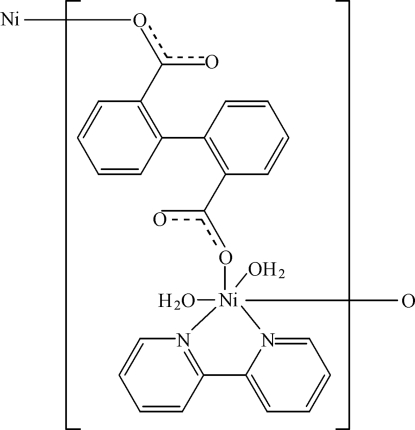


## Experimental

### 

#### Crystal data

[Ni(C_14_H_8_O_4_)(C_10_H_8_N_2_)(H_2_O)_2_]
*M* *_r_* = 491.11
Orthorhombic, 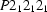

*a* = 10.9087 (15) Å
*b* = 11.214 (2) Å
*c* = 18.129 (3) Å
*V* = 2217.6 (6) Å^3^
*Z* = 4
Mo *K*α radiation
μ = 0.92 mm^−1^
*T* = 296 (2) K
0.42 × 0.27 × 0.19 mm

#### Data collection

Bruker APEXII CCD diffractometer
Absorption correction: multi-scan (*SADABS*; Bruker, 2001 ▶) *T*_min_ = 0.699, *T*_max_ = 0.845
11746 measured reflections
3953 independent reflections
3351 reflections with *I* > 2σ(*I*)
*R*_int_ = 0.033

#### Refinement

*R*[*F* ^2^ > 2σ(*F* ^2^)] = 0.034
*wR*(*F* ^2^) = 0.088
*S* = 1.00
3953 reflections
310 parameters
H atoms treated by a mixture of independent and constrained refinement
Δρ_max_ = 0.21 e Å^−3^
Δρ_min_ = −0.30 e Å^−3^
Absolute structure: Flack (1983 ▶), 1694 Friedel pairs
Flack parameter: 0.042 (16)

### 

Data collection: *APEX2* (Bruker, 2004 ▶); cell refinement: *SAINT-Plus* (Bruker, 2001 ▶); data reduction: *SAINT-Plus*; program(s) used to solve structure: *SHELXS97* (Sheldrick, 2008 ▶); program(s) used to refine structure: *SHELXL97* (Sheldrick, 2008 ▶); molecular graphics: *SHELXTL* (Sheldrick, 2008 ▶); software used to prepare material for publication: *SHELXTL*.

## Supplementary Material

Crystal structure: contains datablocks I, global. DOI: 10.1107/S1600536808036866/bi2317sup1.cif

Structure factors: contains datablocks I. DOI: 10.1107/S1600536808036866/bi2317Isup2.hkl

Additional supplementary materials:  crystallographic information; 3D view; checkCIF report

## Figures and Tables

**Table 1:** Hydrogen-bond geometry (Å, °)

*D*—H⋯*A*	*D*—H	H⋯*A*	*D*⋯*A*	*D*—H⋯*A*
O5—H1*W*⋯*Cg*1	0.86 (2)	2.91	3.741 (3)	163
O5—H2*W*⋯O2^i^	0.85 (2)	1.90 (2)	2.740 (3)	169 (3)
O6—H3*W*⋯O4^i^	0.81 (2)	1.91 (2)	2.676 (4)	158 (4)
O6—H4*W*⋯O2	0.82 (2)	1.98 (2)	2.790 (3)	167 (4)

Symmetry code: (i) 
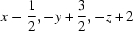
. *Cg*1 is the centroid of the C2–C7 benzene ring.

**Fig. 1.**
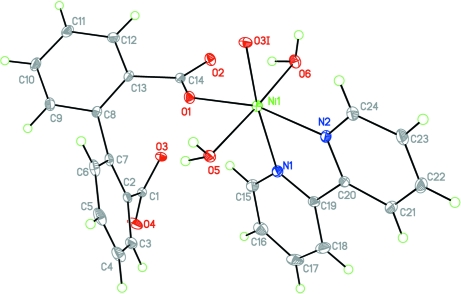
Asymmetric unit of the title compound showing displacement ellipsoids at 30% for non-H atoms.

**Fig. 2.**
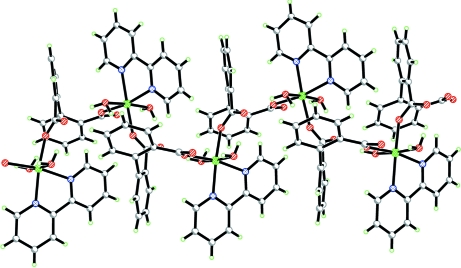
1-D coordination polymer running along the [100] direction.

**Fig. 3.**
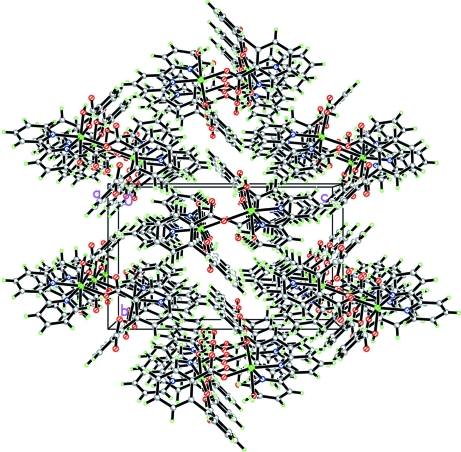
View of the packing along the a axis.

## supplementary crystallographic information

### Comment

2,2'-Biphenyldicarboxylic acid (H_2_dpa) has been demonstrated to be a useful ligand for constructing metal-organic frameworks (Rueff *et al., *2003; Wang *et al., *2006; Xu *et al., *2006). The title compound is a Ni^II^ coordination polymer in which 2,2'-biphenyldicarboxylate (2,2'-dpa) acts as a bridging ligand.

The asymmetric unit (Fig. 1) contains one Ni^II^ atom coordinated by one 2,2'-bipyridine ligand, 2,2'-dpa and two water molecules. The Ni^II^ atom is hexacoordinated in a slightly distorted octahedral geometry by two water molecules, two N atoms from 2,2'-bipyridine, and two O atoms from carboxylate groups of two 2,2'-dpa. The 2,2'-dpa ligand acts as a bridge to link two neighboring Ni^II^ atoms, forming a 1-D coordination polymer along [100] (Fig. 2). Hydrogen bonds from the coordinated water molecules and the O atoms of the carboxylate groups are formed within the same coordination polymer (Fig. 3). One water molecule also forms an O—H···πi interaction to the neighbouring benzene ring of 2,2'-dpa.

### Experimental

A mixture of nickel(II) chloride hexahydrate (0.1 mmol), 2,2'-bipyridine (0.1 mmol), biphenyl-2,2'-dicarboxylic acid (0.2 mmol) and H_2_O (16 ml) in a 25 ml Teflon-lined stainless steel autoclave was kept at 463 K for five days. Green crystals were obtained after cooling to room temperature with a yield of 12%. Elemental analysis calculated: C 58.64, H 4.89, N 5.70%; found: C 58.62, H 4.86, N 5.65%.

### Refinement

H atoms of the water molecules were located from difference Fourier maps and refined freely with *U*_iso_(H) = 1.2*U*_eq_(O). All other H atoms were placed in calculated positions with C—H = 0.93 Å and allowed to ride with *U*_iso_(H) = 1.2*U*_eq_(C).

### Crystal data

**Table:**

[Ni(C_14_H_8_O_4_)(C_10_H_8_N_2_)(H_2_O)_2_]	*F*_000_ = 1016
*M**_r_* = 491.11	*D*_x_ = 1.471 Mg m^−^^3^
Orthorhombic, *P*2_1_2_1_2_1_	Mo *K*α radiation λ = 0.71073 Å
Hall symbol: P 2ac 2ab	Cell parameters from 3953 reflections
*a* = 10.9087 (15) Å	θ = 2.1–25.1º
*b* = 11.214 (2) Å	µ = 0.92 mm^−^^1^
*c* = 18.129 (3) Å	*T* = 296 (2) K
*V* = 2217.6 (6) Å^3^	Block, green
*Z* = 4	0.42 × 0.27 × 0.19 mm

### Data collection

**Table:**

Bruker APEXII CCD diffractometer	3953 independent reflections
Radiation source: fine-focus sealed tube	3351 reflections with *I* > 2σ(*I*)
Monochromator: graphite	*R*_int_ = 0.033
*T* = 296(2) K	θ_max_ = 25.1º
φ and ω scans	θ_min_ = 2.1º
Absorption correction: multi-scan(SADABS; Bruker, 2001)	*h* = −13→12
*T*_min_ = 0.699, *T*_max_ = 0.845	*k* = −9→13
11746 measured reflections	*l* = −20→21

### Refinement

**Table:**

Refinement on *F*^2^	Hydrogen site location: inferred from neighbouring sites
Least-squares matrix: full	H atoms treated by a mixture of independent and constrained refinement
*R*[*F*^2^ > 2σ(*F*^2^)] = 0.034	*w* = 1/[σ^2^(*F*_o_^2^) + (0.051*P*)^2^] where *P* = (*F*_o_^2^ + 2*F*_c_^2^)/3
*wR*(*F*^2^) = 0.088	(Δ/σ)_max_ < 0.001
*S* = 1.00	Δρ_max_ = 0.21 e Å^−^^3^
3953 reflections	Δρ_min_ = −0.30 e Å^−^^3^
310 parameters	Extinction correction: none
Primary atom site location: structure-invariant direct methods	Absolute structure: Flack (1983), 1694 Friedel pairs
Secondary atom site location: difference Fourier map	Flack parameter: 0.042 (16)

### Special details

**Table:**

Geometry. All e.s.d.'s (except the e.s.d. in the dihedral angle between two l.s. planes) are estimated using the full covariance matrix. The cell e.s.d.'s are taken into account individually in the estimation of e.s.d.'s in distances, angles and torsion angles; correlations between e.s.d.'s in cell parameters are only used when they are defined by crystal symmetry. An approximate (isotropic) treatment of cell e.s.d.'s is used for estimating e.s.d.'s involving l.s. planes.
Refinement. Refinement of *F*^2^ against ALL reflections. The weighted *R*-factor *wR* and goodness of fit *S* are based on *F*^2^, conventional *R*-factors *R* are based on *F*, with *F* set to zero for negative *F*^2^. The threshold expression of *F*^2^ > σ(*F*^2^) is used only for calculating *R*-factors(gt) *etc*. and is not relevant to the choice of reflections for refinement. *R*-factors based on *F*^2^ are statistically about twice as large as those based on *F*, and *R*- factors based on ALL data will be even larger.

### Fractional atomic coordinates and isotropic or equivalent isotropic displacement parameters (Å^2^)

**Table:**

	*x*	*y*	*z*	*U*_iso_*/*U*_eq_	
Ni1	0.24756 (4)	0.69154 (3)	0.898676 (19)	0.03527 (12)	
C1	0.5625 (3)	1.0010 (3)	1.06148 (16)	0.0367 (7)	
C2	0.4305 (3)	1.0354 (3)	1.03948 (16)	0.0377 (8)	
C3	0.4167 (4)	1.1232 (3)	0.98610 (18)	0.0525 (9)	
H3	0.4864	1.1582	0.9661	0.063*	
C4	0.3031 (5)	1.1602 (4)	0.9617 (2)	0.0673 (14)	
H4	0.2977	1.2188	0.9255	0.081*	
C5	0.1971 (4)	1.1108 (4)	0.9905 (2)	0.0616 (12)	
H5	0.1208	1.1353	0.9735	0.074*	
C6	0.2059 (3)	1.0243 (3)	1.04525 (19)	0.0515 (10)	
H6	0.1351	0.9918	1.0656	0.062*	
C7	0.3233 (3)	0.9853 (3)	1.07010 (16)	0.0386 (8)	
C8	0.3257 (3)	0.9030 (3)	1.13631 (16)	0.0360 (7)	
C9	0.3102 (3)	0.9525 (3)	1.20745 (18)	0.0492 (9)	
H9	0.2988	1.0345	1.2110	0.059*	
C10	0.3108 (4)	0.8874 (4)	1.27158 (19)	0.0552 (10)	
H10	0.2954	0.9239	1.3167	0.066*	
C11	0.3343 (4)	0.7687 (4)	1.26763 (18)	0.0553 (10)	
H11	0.3383	0.7222	1.3101	0.066*	
C12	0.3524 (3)	0.7181 (3)	1.19772 (17)	0.0469 (8)	
H12	0.3706	0.6372	1.1950	0.056*	
C13	0.3445 (3)	0.7820 (3)	1.13252 (16)	0.0356 (7)	
C14	0.3500 (3)	0.7168 (3)	1.05824 (16)	0.0342 (7)	
C15	0.4937 (3)	0.8346 (3)	0.8927 (2)	0.0509 (9)	
H15	0.4958	0.8224	0.9435	0.061*	
C16	0.5799 (3)	0.9085 (3)	0.8606 (2)	0.0608 (10)	
H16	0.6392	0.9457	0.8894	0.073*	
C17	0.5771 (4)	0.9267 (4)	0.7847 (3)	0.0678 (12)	
H17	0.6337	0.9772	0.7625	0.081*	
C18	0.4900 (4)	0.8695 (4)	0.7424 (2)	0.0596 (10)	
H18	0.4877	0.8802	0.6915	0.071*	
C19	0.4047 (3)	0.7949 (3)	0.77769 (18)	0.0401 (7)	
C20	0.3066 (3)	0.7286 (3)	0.73743 (17)	0.0383 (8)	
C21	0.3005 (4)	0.7230 (3)	0.65934 (18)	0.0527 (10)	
H21	0.3595	0.7609	0.6306	0.063*	
C22	0.2058 (4)	0.6605 (3)	0.6264 (2)	0.0587 (11)	
H22	0.2007	0.6555	0.5753	0.070*	
C23	0.1192 (4)	0.6058 (3)	0.66986 (18)	0.0525 (10)	
H23	0.0547	0.5638	0.6486	0.063*	
C24	0.1297 (3)	0.6143 (3)	0.74713 (18)	0.0451 (8)	
H24	0.0705	0.5780	0.7764	0.054*	
N1	0.4065 (2)	0.7797 (2)	0.85219 (14)	0.0402 (7)	
N2	0.2231 (2)	0.6733 (2)	0.78055 (13)	0.0363 (6)	
O1	0.28142 (19)	0.75413 (19)	1.00612 (11)	0.0407 (6)	
O2	0.4185 (2)	0.6272 (2)	1.05422 (12)	0.0451 (6)	
O3	0.58196 (19)	0.89368 (18)	1.07528 (12)	0.0385 (5)	
O4	0.6422 (2)	1.0789 (2)	1.06222 (17)	0.0670 (8)	
O5	0.1519 (2)	0.85857 (18)	0.89069 (12)	0.0394 (5)	
H1W	0.187 (2)	0.895 (3)	0.9264 (15)	0.047*	
H2W	0.0764 (16)	0.858 (3)	0.9025 (17)	0.047*	
O6	0.3552 (2)	0.5345 (2)	0.91659 (13)	0.0466 (6)	
H3W	0.302 (3)	0.484 (3)	0.9225 (18)	0.056*	
H4W	0.385 (3)	0.557 (3)	0.9558 (13)	0.056*	

### Atomic displacement parameters (Å^2^)

**Table:**

	*U*^11^	*U*^22^	*U*^33^	*U*^12^	*U*^13^	*U*^23^
Ni1	0.0390 (2)	0.0344 (2)	0.0325 (2)	−0.0010 (2)	0.0015 (2)	−0.00163 (16)
C1	0.047 (2)	0.0325 (18)	0.0310 (16)	0.0033 (16)	0.0056 (14)	0.0009 (14)
C2	0.058 (2)	0.0275 (16)	0.0275 (15)	0.0093 (16)	−0.0028 (15)	−0.0034 (13)
C3	0.080 (3)	0.0377 (19)	0.0401 (19)	0.006 (2)	−0.0049 (19)	0.0003 (17)
C4	0.114 (4)	0.042 (2)	0.045 (2)	0.026 (2)	−0.033 (2)	−0.0035 (18)
C5	0.080 (3)	0.049 (2)	0.056 (2)	0.034 (2)	−0.032 (2)	−0.015 (2)
C6	0.054 (2)	0.052 (2)	0.048 (2)	0.0185 (18)	−0.0131 (16)	−0.0215 (18)
C7	0.049 (2)	0.0350 (18)	0.0319 (16)	0.0132 (15)	−0.0035 (15)	−0.0093 (14)
C8	0.0320 (17)	0.0416 (18)	0.0343 (16)	0.0010 (14)	0.0026 (14)	−0.0022 (14)
C9	0.056 (2)	0.046 (2)	0.045 (2)	0.0093 (17)	0.0060 (17)	−0.0082 (17)
C10	0.065 (2)	0.068 (3)	0.0335 (18)	−0.004 (2)	0.0110 (17)	−0.0084 (18)
C11	0.071 (3)	0.065 (3)	0.0296 (17)	−0.011 (2)	0.0051 (18)	0.0021 (17)
C12	0.059 (2)	0.0417 (19)	0.0402 (18)	−0.0088 (17)	−0.0019 (17)	0.0041 (16)
C13	0.0349 (17)	0.0436 (19)	0.0283 (15)	−0.0034 (15)	0.0040 (13)	−0.0020 (14)
C14	0.0370 (17)	0.0334 (18)	0.0321 (16)	−0.0062 (15)	0.0009 (14)	−0.0021 (14)
C15	0.0413 (19)	0.054 (2)	0.057 (2)	−0.0117 (16)	0.0082 (18)	−0.0048 (19)
C16	0.043 (2)	0.053 (2)	0.087 (3)	−0.0107 (19)	0.007 (2)	−0.002 (2)
C17	0.048 (2)	0.054 (2)	0.102 (3)	−0.013 (2)	0.015 (2)	0.019 (2)
C18	0.061 (2)	0.056 (2)	0.062 (2)	−0.002 (2)	0.016 (2)	0.018 (2)
C19	0.0407 (17)	0.0313 (17)	0.0482 (19)	0.0054 (15)	0.0106 (15)	0.0042 (15)
C20	0.0513 (19)	0.0280 (16)	0.0356 (17)	0.0079 (15)	0.0065 (15)	0.0024 (14)
C21	0.078 (3)	0.043 (2)	0.0376 (18)	0.0082 (19)	0.0099 (18)	0.0045 (16)
C22	0.096 (3)	0.050 (2)	0.0305 (17)	0.018 (2)	−0.0062 (19)	0.0007 (17)
C23	0.072 (3)	0.041 (2)	0.044 (2)	0.0062 (19)	−0.0159 (18)	−0.0114 (18)
C24	0.053 (2)	0.042 (2)	0.0401 (19)	−0.0008 (17)	−0.0012 (16)	−0.0061 (16)
N1	0.0388 (15)	0.0395 (15)	0.0423 (16)	−0.0044 (13)	0.0074 (12)	−0.0037 (13)
N2	0.0432 (16)	0.0361 (14)	0.0295 (12)	0.0032 (12)	0.0015 (11)	−0.0020 (11)
O1	0.0520 (14)	0.0380 (12)	0.0322 (11)	0.0053 (10)	−0.0061 (10)	−0.0038 (9)
O2	0.0518 (14)	0.0400 (13)	0.0436 (13)	0.0029 (12)	−0.0086 (11)	−0.0076 (11)
O3	0.0403 (12)	0.0297 (12)	0.0456 (12)	0.0018 (10)	−0.0019 (10)	0.0082 (10)
O4	0.0539 (15)	0.0333 (14)	0.114 (2)	−0.0040 (13)	0.0030 (16)	0.0071 (15)
O5	0.0418 (12)	0.0328 (12)	0.0436 (13)	0.0019 (10)	0.0014 (11)	−0.0006 (10)
O6	0.0465 (15)	0.0415 (14)	0.0519 (15)	0.0074 (11)	−0.0036 (12)	−0.0072 (12)

### Geometric parameters (Å, °)

**Table:**

Ni1—O3^i^	2.098 (2)	C13—C14	1.533 (4)
Ni1—O1	2.103 (2)	C14—O2	1.255 (4)
Ni1—O6	2.142 (2)	C14—O1	1.276 (3)
Ni1—O5	2.149 (2)	C15—N1	1.351 (4)
Ni1—N1	2.166 (3)	C15—C16	1.383 (5)
Ni1—N2	2.168 (2)	C15—H15	0.930
C1—O4	1.233 (4)	C16—C17	1.391 (6)
C1—O3	1.247 (4)	C16—H16	0.930
C1—C2	1.543 (5)	C17—C18	1.379 (6)
C2—C3	1.388 (5)	C17—H17	0.930
C2—C7	1.412 (5)	C18—C19	1.406 (5)
C3—C4	1.380 (6)	C18—H18	0.930
C3—H3	0.930	C19—N1	1.361 (4)
C4—C5	1.385 (6)	C19—C20	1.493 (5)
C4—H4	0.930	C20—N2	1.352 (4)
C5—C6	1.391 (5)	C20—C21	1.419 (4)
C5—H5	0.930	C21—C22	1.385 (6)
C6—C7	1.427 (4)	C21—H21	0.930
C6—H6	0.930	C22—C23	1.374 (5)
C7—C8	1.514 (4)	C22—H22	0.930
C8—C13	1.374 (5)	C23—C24	1.409 (5)
C8—C9	1.414 (4)	C23—H23	0.930
C9—C10	1.373 (5)	C24—N2	1.357 (4)
C9—H9	0.930	C24—H24	0.930
C10—C11	1.358 (6)	O3—Ni1^ii^	2.098 (2)
C10—H10	0.930	O5—H1W	0.86 (2)
C11—C12	1.402 (5)	O5—H2W	0.85 (2)
C11—H11	0.930	O6—H3W	0.81 (2)
C12—C13	1.385 (4)	O6—H4W	0.82 (2)
C12—H12	0.930		
			
O3^i^—Ni1—O1	95.44 (8)	C8—C13—C12	118.5 (3)
O3^i^—Ni1—O6	93.63 (9)	C8—C13—C14	121.3 (3)
O1—Ni1—O6	92.16 (9)	C12—C13—C14	120.1 (3)
O3^i^—Ni1—O5	89.65 (8)	O2—C14—O1	124.7 (3)
O1—Ni1—O5	81.77 (8)	O2—C14—C13	117.1 (3)
O6—Ni1—O5	173.36 (9)	O1—C14—C13	118.1 (3)
O3^i^—Ni1—N1	169.97 (9)	N1—C15—C16	121.5 (4)
O1—Ni1—N1	93.86 (9)	N1—C15—H15	119.2
O6—Ni1—N1	89.75 (10)	C16—C15—H15	119.2
O5—Ni1—N1	87.97 (9)	C15—C16—C17	119.3 (4)
O3^i^—Ni1—N2	94.20 (9)	C15—C16—H16	120.3
O1—Ni1—N2	165.48 (9)	C17—C16—H16	120.4
O6—Ni1—N2	98.04 (9)	C18—C17—C16	119.8 (4)
O5—Ni1—N2	87.46 (9)	C18—C17—H17	120.1
N1—Ni1—N2	75.96 (10)	C16—C17—H17	120.1
O4—C1—O3	124.2 (3)	C17—C18—C19	118.7 (4)
O4—C1—C2	118.9 (3)	C17—C18—H18	120.7
O3—C1—C2	116.9 (3)	C19—C18—H18	120.7
C3—C2—C7	117.8 (3)	N1—C19—C18	121.1 (3)
C3—C2—C1	117.3 (3)	N1—C19—C20	115.7 (3)
C7—C2—C1	124.9 (3)	C18—C19—C20	123.2 (3)
C4—C3—C2	122.3 (4)	N2—C20—C21	121.7 (3)
C4—C3—H3	118.8	N2—C20—C19	115.4 (3)
C2—C3—H3	118.8	C21—C20—C19	122.9 (3)
C5—C4—C3	120.5 (3)	C22—C21—C20	119.2 (4)
C5—C4—H4	119.7	C22—C21—H21	120.4
C3—C4—H4	119.7	C20—C21—H21	120.4
C4—C5—C6	119.4 (3)	C23—C22—C21	119.5 (3)
C4—C5—H5	120.3	C23—C22—H22	120.3
C6—C5—H5	120.3	C21—C22—H22	120.3
C5—C6—C7	120.0 (4)	C22—C23—C24	119.0 (3)
C5—C6—H6	120.0	C22—C23—H23	120.5
C7—C6—H6	120.0	C24—C23—H23	120.5
C2—C7—C6	119.8 (3)	N2—C24—C23	122.5 (3)
C2—C7—C8	122.7 (3)	N2—C24—H24	118.7
C6—C7—C8	116.9 (3)	C23—C24—H24	118.7
C13—C8—C9	116.8 (3)	C15—N1—C19	119.5 (3)
C13—C8—C7	124.4 (3)	C15—N1—Ni1	124.1 (2)
C9—C8—C7	118.8 (3)	C19—N1—Ni1	115.6 (2)
C10—C9—C8	124.3 (3)	C20—N2—C24	118.1 (3)
C10—C9—H9	117.9	C20—N2—Ni1	116.4 (2)
C8—C9—H9	117.8	C24—N2—Ni1	125.4 (2)
C11—C10—C9	118.5 (3)	C14—O1—Ni1	132.81 (19)
C11—C10—H10	120.7	C1—O3—Ni1^ii^	129.1 (2)
C9—C10—H10	120.8	Ni1—O5—H1W	99 (2)
C10—C11—C12	118.1 (3)	Ni1—O5—H2W	117 (2)
C10—C11—H11	121.0	H1W—O5—H2W	104 (2)
C12—C11—H11	120.9	Ni1—O6—H3W	102 (3)
C13—C12—C11	123.6 (3)	Ni1—O6—H4W	95 (3)
C13—C12—H12	118.2	H3W—O6—H4W	112 (3)
C11—C12—H12	118.2		

Symmetry codes: (i) *x*−1/2, −*y*+3/2, −*z*+2; (ii) *x*+1/2, −*y*+3/2, −*z*+2.

### Hydrogen-bond geometry (Å, °)

**Table:**

*D*—H···*A*	*D*—H	H···*A*	*D*···*A*	*D*—H···*A*
O5—H1W···Cg1	0.86 (2)	2.91	3.741 (3)	163
O5—H2W···O2^i^	0.85 (2)	1.90 (2)	2.740 (3)	169 (3)
O6—H3W···O4^i^	0.81 (2)	1.91 (2)	2.676 (4)	158 (4)
O6—H4W···O2	0.82 (2)	1.98 (2)	2.790 (3)	167 (4)

Symmetry codes: (i) *x*−1/2, −*y*+3/2, −*z*+2.

## References

[bb1] Bruker (2001). *SADABS *and *SAINT-Plus* Bruker AXS Inc., Madison, Wisconsin, USA.

[bb2] Bruker (2004). *APEX2* Bruker AXS Inc., Madison, Wisconsin, USA.

[bb3] Flack, H. D. (1983). *Acta Cryst.* A**39**, 876–881.

[bb4] Rueff, J.-M., Pillet, S., Bonaventure, G., Souhassou, M. & Rabu, P. (2003). *Eur. J. Inorg. Chem.* pp. 4173–4178.

[bb5] Sheldrick, G. M. (2008). *Acta Cryst.* A**64**, 112–122. 10.1107/S0108767307043930 18156677

[bb6] Wang, R.-H., Gong, Y.-Q., Han, L., Yuan, D.-Q., Lou, B.-Y., Wu, B.-L. & Hong, M.-C. (2006). *J. Mol. Struct.* **784**, 1–6.

[bb7] Xu, X.-X., Lu, Y., Wang, E.-B., Ma, Y. & Bai, X.-L. (2006). *Cryst. Growth Des.* **6**, 2029–2035.

